# Effects of real‐time VR clinical practice on reducing the stigma toward dementia among students of occupational therapy: A randomized controlled trial

**DOI:** 10.1002/pcn5.160

**Published:** 2023-12-03

**Authors:** Keita Ueno, Hiroyuki Tanaka, Kazuyuki Niki, Masaya Ueda, Ayumi Tanaka, Katsushi Yokoi, Yasuo Naito, Ryouhei Ishii

**Affiliations:** ^1^ Department of Occupational Therapy, Graduate School of Rehabilitation Science Osaka Metropolitan University Habikino Japan; ^2^ Department of Clinical Pharmacy Research and Education, Graduate School of Pharmaceutical Sciences Osaka University Suita Japan; ^3^ Department of Rehabilitation Geriatric Health Service Facility Vansanku, Kaseikai Osaka Japan; ^4^ Department of Psychiatry, Graduate School of Medicine Osaka University Suita Japan

**Keywords:** dementia, occupational therapy, online education, real‐time virtual reality, stigma

## Abstract

**Aim:**

This study aimed to examine the effects of real‐time online clinical practice using real‐time virtual reality (VR) compared with 2D PC screening on reducing stigma toward dementia, and to investigate the feasibility of online clinical practice using VR.

**Methods:**

A single‐center, open‐label, randomized controlled trial was conducted. Occupational therapy students were randomized to view occupational therapy evaluation screens for dementia patients using a VR headset or 2D monitor. The Attitudes Toward Dementia Scale (ADS), the Dementia Knowledge Scale (DKS), and Images of the Elderly with Dementia (IED) were assessed before and after the intervention. The level of clinical practice satisfaction and the System Usability Scale (SUS) were also assessed.

**Results:**

The number of subjects in the intervention and control groups was 10 and 9, respectively. In ADS scores and IED, the main effect was shown in both groups and did not show interactions. In DKS scores, the main effect and interaction were not shown. The VR headset tended to be more usable than the 2D monitor in terms of usability. Satisfaction ratings indicated the characteristics of a realistic clinical experience through real‐time VR viewing.

**Conclusion:**

Real‐time VR and 2D online clinical practice could reduce the stigma toward dementia, but there were no significant differences between the types. The real‐time VR experience was more similar to actual clinical practice than a 2D PC screening due to the sense of immersion, but issues in blinding and lack of audio and video quality were found.

## Graphical abstract

This study shows that real‐time, online clinical practice experiences, including real‐time virtual reality (VR), can positively change attitudes toward dementia. Online clinical practice can be experienced at a distance, and a large group of people can experience the same scene. Real‐time VR has higher usability and the experience is more similar to actual clinical practice than a 2D PC screen due to the amount of information provided by the 360‐degree images and the sense of immersion.

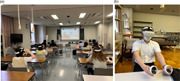

## INTRODUCTION

As the number of older people increases, medical and health science students, such as occupational therapy students, will have more opportunities to meet people with dementia. Dementia is often the target of discrimination and stigma. Stigma against people with mental health conditions, especially public and interpersonal stigma, refers to the link between stereotypes, negative attitudes, and discrimination against people with mental health conditions in society.^1^ For people with dementia, this can lead to isolation, reduced quality of life, low self‐esteem and depression.^2^ Reducing stigma in medical education is therefore important.

It is well known that stigma is reduced by face‐to‐face contact.^3^ However, clinical practice has been restricted by the outbreak of COVID‐19 and many medical students have had little experience of contact with people with dementia, reducing opportunities to reduce stigma. In this restricted educational environment, the development of telecommunications technology has enabled online lectures and skills training.^4^, ^5^ We reported that stigma, assessed as attitudes and images of mental disorders, was improved in occupational therapy students by observing group therapy for people with a mental disorder using the Zoom application on PCs.^6^ However, online clinical practice using PCs is less immersive than face‐to‐face practice and the information, such as the atmosphere of the clinical setting and the condition of the subject, is limited, which is an issue that needs to be addressed.

More recently, virtual reality (VR) technology has been used in medical and educational settings to address these issues.^7^, ^8^ Omori et al.^9^ investigated the effectiveness of VR as a learning tool for improving infection‐control procedures and showed that VR learning may improve learning effect, learner satisfaction, motivation, and concentration. Wu et al.^10^ conducted a meta‐analysis on the learning performance effectiveness of VR using head‐mounted displays and found that immersive learning is more beneficial for young learners and the field of science education compared to traditional real‐world education. However, education using VR systems has the following problems: (1) continuous training and financial support for teachers and instructors is necessary, and (2) reduction in concentration due to simulation sickness of students.^8^ Additionally, common VR systems under a normal Internet environment have several disadvantages, including time lag in communications for more than a few seconds.

The recent emergence of new VR systems capable of high‐speed, low‐latency communication has made real‐time communication possible even in the VR world. AVATOUR is one of these solutions that allows 360‐degree spatial images to be shared and interactive communication in real‐time (Avatour Technologies, Inc.). Using AVATOUR, images captured by a 360‐degree camera can be distributed via a smartphone and viewed via the Internet using VR headsets.

Therefore, we hypothesized that real‐time online clinical practice using real‐time VR would reduce dementia stigma more than 2D online practice. However, it is necessary to examine the safety and usability of real‐time VR in a pilot study. In addition, although high‐speed, low‐latency communication is available, there is a need to investigate what impression the degree of accuracy of the images and sound will have on students. We also need to identify measures that can accurately assess whether online clinical practice improves stigma against dementia. The aim of this study was to investigate the effects of real‐time online clinical practice using real‐time VR compared with 2D PC screening on reducing stigma toward dementia in occupational therapy students, and to examine the feasibility of online clinical practice using VR, the relevance of assessment choices, and the ease of use of the device.

## METHODS

### Study design

A single‐center, open‐label, randomized controlled trial with a pre–post design was conducted. Participants were randomly assigned to an intervention group and a control group. The intervention group observed and communicated with a patient with dementia using VR, while the control group used a 2D projected monitor on a screen. Participant recruitment and intervention were conducted in July 2022.

### Participants

Participants were recruited from third‐year students of the occupational therapy major, School of Comprehensive Rehabilitation, Osaka Metropolitan University (*n* = 26). Inclusion criteria were set at the grade level considered most appropriate for the educational program of the course of occupational therapy major at Osaka Metropolitan University; they did not hold medical or social work qualifications and had not experienced long‐term clinical practice. Exclusion criteria were set to those who had received similar education or training on or off campus by VR. Participants were also excluded from data analysis if they refused consent, withdrew consent, or could not continue because of VR sickness or other adverse events. Before participating in this study, all participants were informed orally and in writing of the purpose, content, and handling of personal information of the study, and gave their signed consent.

### Allocation to intervention group (real‐time VR) and control group (2D monitor)

Each participant was allocated so that the real‐time VR group and the 2D monitor group had the same number of students, using a computer‐generated random number sequence. We used permuted blocked randomization, with block sizes of two. The results of the allocation were not blinded to the participants or the intervention providers who set up the practice setting at the university. The first author who conducted the data analysis was blinded from the allocation results.

### Devices used in the intervention

The intervention group used a VR headset (Meta Quest 2, Meta Platforms, Inc.) with a 360‐degree video real‐time distribution service (AVATOUR, NTT Communications Corporation) installed.^11^ AVATOUR is a solution that allows 360‐degree spatial images to be shared in real‐time (Avatour Technologies, Inc.). Using AVATOUR, images captured by a 360‐degree camera can be distributed via a smartphone and viewed via the Internet using a VR headset. The speakers and microphone system built into the VR headset were used, and the camera was not used because it was not necessary to transmute the students' image to the collaborating institution.

The control group used the PC browser version of AVATOUR. A projector was connected to a PC to display the video streamed from the collaborating institution, and the control group watched the projected monitor on a screen in the classroom.

### Environmental setting at the university

The intervention group and control group were arranged to receive the online clinical training at the same time in separate rooms next to each other at the university to avoid audio mix‐ups.

### Environmental setting at the collaborating institution

The collaborating site was a geriatric healthcare institution in Osaka, Japan. As the environmental setting, observed subjects, occupational therapists working at the facility, and a researcher entered a private room in the facility. A 360‐degree camera (insta360 ONE RS, Shenzhen Arashi Vision, Inc.) was connected to the smartphone (Xperia 1 II SO‐51A, Sony Inc.) with AVATOUR installed, and placed at a point where the observed subject and occupational therapist could be seen. Wireless microphone speakers (OfficeCore M2, Shenzhen eMeet technology Co., Ltd.) were used to make it easier for the students to hear the conversation between the observed subject and the occupational therapist.

One elderly patient with dementia who was institutionalized at the site participated in the online clinical practice. The observed subject was female, 88 years old and diagnosed with dementia. We assessed her stage of dementia as mild by applying the Clinical Dementia Rating. She had short‐term memory impairment and disorientation regarding the date and year. In terms of physical abilities, she could perform most of her basic activities of daily living independently, such as toileting, although she spent most of her daily time in a wheelchair.

### Procedure

**Figure 1:**
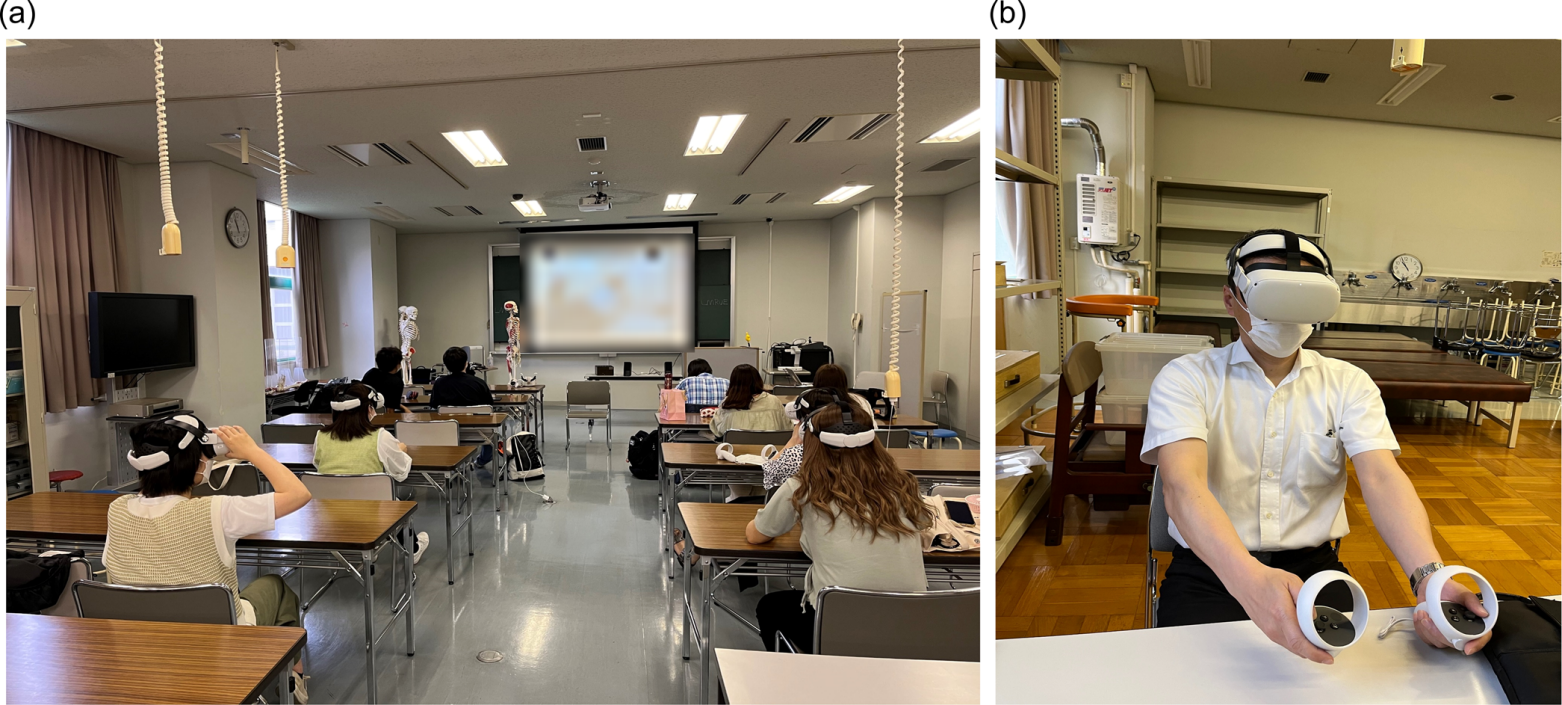
The intervention group and the control group were arranged to receive online clinical practice at the same time in separate rooms. (a) Location of intervention. (b) A participant wearing a VR headset.

Participants who signed the informed consent form received a 30‐min instruction on how to use the VR and AVATOUR 1 week prior to the intervention. On the day of the intervention, each group was observed for 20 min in an occupational therapy session. The session consisted of the Mini‐Mental State Examination, range of motion measurement, Manual Muscle Test, and Hearing of Life History. Afterwards, a 20‐min question‐and‐answer session was held with the observed subject and the occupational therapist regarding the content of the occupational therapy session. The intervention group participated with real‐time VR headsets, while the control group participated with a 2D projected monitor on a screen (Figure 1).

### Assessments (outcome measure)

Assessments were conducted at two time‐points: just before the intervention and just after the intervention ended. Demographics including participants' age, sex, experience and frequency of contact with dementia patients, cohabitation, and concern about dementia, were assessed before the intervention. The effect of the intervention on the stigma toward dementia was assessed using the Attitudes Toward Dementia Scale (ADS), the Dementia Knowledge Scale (DKS), and Images of the Elderly with Dementia (IED). In addition, the usability of this online practice with real‐time VR and 2D monitor was assessed by the System Usability Scale (SUS). The level of class satisfaction in this clinical practice was assessed by a questionnaire.

The ADS was set as the primary outcome, and other assessments were set as secondary outcomes. The ADS is one of the most widely used scales in Japan to measure attitudes and self‐perceptions regarding dementia, with reliability and validity assured.^12^ It is a four‐point Likert scale ranging from *Disagree* to *Agree*. Total scores range from 15 to 60, and higher scores indicate better attitudes toward dementia. In assessing stigma, the most used measure is that of an individual's attitudes toward the disease. The DKS is also one of the most widely used scales in Japan to assess knowledge about dementia, with proven reliability and validity.^12^ The scale is scored on a scale of 0–15, with higher scores indicating greater knowledge of dementia. Knowledge of the disease is also a factor in determining attitudes.

Since the image of the elderly with dementia is an important factor in forming attitudes, which is the primary outcome, the image‐rating method used in a previous study^12^ was also used in this study. The images were rated on a 5‐point scale with 12 items of positive and negative images, such as “bright/dark” and “happy/unhappy.” Scores ranged from 12 to 60, with higher scores indicating more positive images of dementia.

The usability of this online clinical practice with real‐time VR and 2D monitor was evaluated by the SUS. The SUS is a tool used to evaluate the usability of products such as websites and interactive voice response systems.^13^ It provides a score from 0 to 100, with higher scores indicating good usability.

Class satisfaction was assessed using 12 items from our university's regular class satisfaction questionnaire and three free descriptive items added by the researchers for this study. The content of the questionnaire consisted of 12 items, including items such as “How much did you concentrate on this online clinical practice compared to your usual class?” It is a six‐point Likert scale with scores ranging from *Strongly disagree* to *Strongly agree*. The other three items are open‐ended descriptions, such as “What were good points about this online clinical practice?” and “What could be improved about the online clinical practice?” To assess safety, any adverse events during the intervention (e.g., VR sickness, neck/head pain, falling out of the chair, etc.) were evaluated.

### Statistical analysis

Participant demographics are compared between the two groups at baseline using the Mann–Whitney *U*‐test or the chi‐square test. The objective of the study was to confirm whether real‐time VR was superior to 2D screening in reducing stigma toward dementia assessed as attitudes. Repeated‐measures two‐way analysis of variance and calculated main effects, interactions, and effect sizes were performed on ADS, DKS, and IED in the two groups. In addition, hierarchical multiple regression analysis was performed to identify influencing factors for ADS, DKS, and IED with each post‐intervention score as the dependent variable and groups in the first step, and sex and each pre‐intervention score in the second step, as independent variables. Variables with variance inflation factors (VIFs) > 10 were excluded from the analysis to avoid multicollinearity. Using Wilcoxon signed‐rank sum test, we calculated effect sizes (r) for ADS, DKS, and IED for all participants, the intervention group, and the control group, respectively.

In the post‐intervention analysis, the Mann–Whitney *U*‐test was performed to examine the difference in the SUS scores between the two groups. Descriptive statistics were performed for the 12 items of class satisfaction.

SPSS Version 28 (IBM Japan, Ltd.) was used for the above statistical analyses, and a significance level of less than 0.05 was adopted. Quantitative text analysis using the KH Coder (!https://khcoder.net/en/) was used to analyze the open‐ended descriptions in three items of the class satisfaction questionnaire. Quantitative content analysis is a method in which researchers make qualitative interpretations of raw words by referring to the results of the quantitative analysis, thus ensuring the reliability of the data.^14^ In this study, we used this approach as an objective and reproducible method, and it could avoid the effect of the subjective perceptions of the researcher. In this analysis, we performed a multivariate analysis on the frequent words in the qualitative data and created a co‐occurrence network to ensure the connections between words in a cluster, which allowed us to search for the concepts and clusters contained in the data.^15^ The Jaccard coefficient, which was used as an indicator of the association between words, indicates a value between 0 and 1, and a value greater than 0.2 is considered to indicate a strong association. A larger circle in the figure indicates a higher frequency of occurrence. Also, A “correspondence analysis” was also conducted to understand the characteristics of the free descriptions of the intervention group (real‐time VR) and the control group. Correspondence analysis was a method of visually understanding the relationship between words that were characteristic of each group. Words that were common to both the groups are concentrated near the origin. The characteristic words of each group were placed far from the origin.^16^

### Ethical consideration

This study was conducted with the approval of the ethics committee of Osaka Metropolitan University Graduate School General Rehabilitation Studies (2022–207). In addition, the registration information for this study can be found at the following URL: https://center6.umin.ac.jp/cgi-open-bin/ctr/ctr_view.cgi?recptno=R00005.

## RESULTS

### Participant demographics

**Figure 2:**
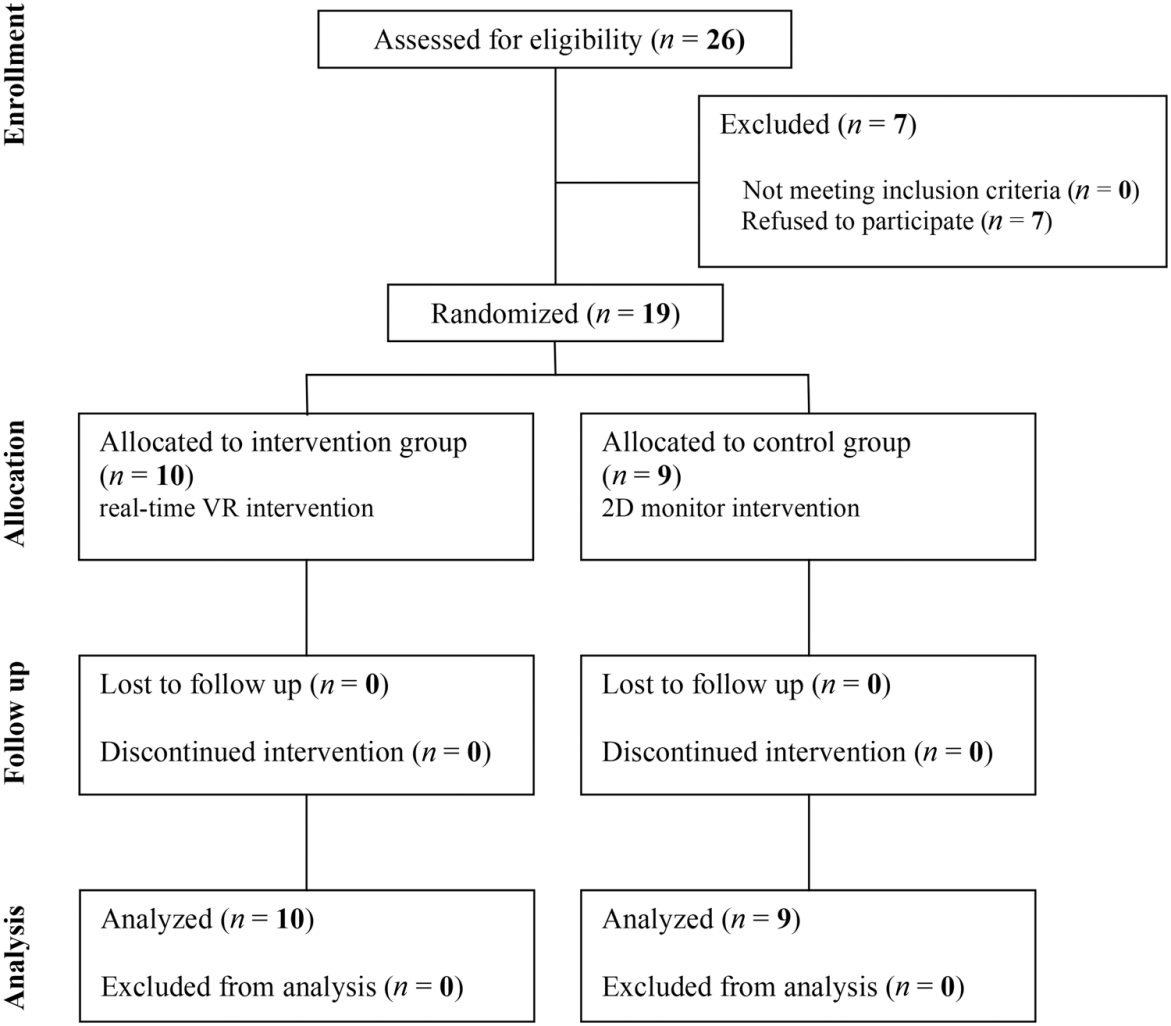
Consolidated Standards of Reporting Trials (CONSORT) diagram of participant flow through this study.

Nineteen students gave signed consent and participated (mean age: 20.5 ± 0.8 years, 3 males, and 16 females) (Figure 2). Subsequently, 10 members of the intervention group (mean age: 20.1 ± 0.3 years, one male, and nine females) and nine members of control group (mean age: 20.9 ± 0.9 years, two males and seven females) were assigned. All subjects experienced no adverse events during the intervention. At baseline, there were no significant differences between the intervention and control groups in terms of age, sex, experience/frequency of contact with dementia patients, cohabitation or level of concern about dementia (Table 1).

**Table 1:** Baseline characteristics of participants.

	Intervention group (*n* = 10)	Control group (*n* = 9)
Mean age (years)	20.1 ± 0.3	20.9 ± 0.9
Sex (Male: Female)	1: 9	2: 7
Experience of contact with dementia patients (Yes: No)	4: 6	5: 4
Living with a dementia patient (Yes: No)	1: 9	0: 9
Frequency of contact with dementia patients: Almost every day: A few times a week: Less than once a week: Not very often: Never	0: 0: 0: 3: 7	0: 0: 1: 0: 8
Concern for Dementia: Yes: Rather yes: Rather no: No	5: 5: 0: 0	5: 3: 1: 0
Dementia Attitude Scale (15–60 points)	44.9 ± 4.3	46.6 ± 4.5
Dementia Knowledge Scale (0–15 points)	13.1 ± 1.1	12.9 ± 2.0
Image for dementia and elderly (12–60 points)	40.7 ± 6.1	36.9 ± 6.5

### Attitudes Toward Dementia Scale

The ADS scores of the intervention group changed from 44.9 ± 4.3 to 47.3 ± 3.3, while those of the control group changed from 46.6 ± 4.5 to 48.6 ± 4.9, indicating the main effect in clinical practice (*F* value = 12.891, *p* = 0.002) and no interactions (*F* value = 0.033, *p* = 0.858) (Table 2). The results of hierarchical multiple regression analysis showed that in the first stage, groups were not associated with post‐intervention ADS score (*β* = −0.146, *p* = 0.550), and in the second stage, pre‐intervention ADS score was significantly associated with post‐intervention ADS score (*β* = 0.789, *p* < 0.001) (Table 3). Wilcoxon signed rank sum test results showed a large effect size for the overall participants (*r* = −0.65), a large effect size for the intervention group (*r* = −0.52), and a medium effect size for the control group (*r* = −0.40).

**Table 2:** Changes in various assessment index scores before and after intervention.

	Intervention group (*n* = 10)		Control group (*n* = 9)		*F* value (Main effect)	*F* value (Interaction)
	Pre	Post	Pre	Post		
ADS	44.9 ± 4.3	47.4 ± 3.3	46.6 ± 4.5	48.6 ± 4.9	12.891**	0.033
DKS	13.1 ± 1.1	12.3 ± 1.7	12.9 ± 2.0	13.3 ± 1.2	0.256	1.561
IED	40.7 ± 6.1	48.5 ± 6.5	36.9 ± 6.5	47.6 ± 8.0	39.029***	0.422

*Note*: Two‐way repeated‐measures analysis of variance.

Abbreviations: ADS, Attitudes Toward Dementia Scale; DKS, Dementia Knowledge Scale; IED, Images of the Elderly with Dementia.

** *p* < 0.01

*** *p* < 0.001.

**Table 3:** Hierarchical multiple regression analysis of online clinical practice using VR or 2D monitors for predicting ADS scores after intervention.

Predictor variables	Adjusted *R* ^2^	*β*	95% CI for B		*t*	*p*‐value
			Lower	Upper		
Crude model	−0.036					0.550
Groups		−0.146	−5.150	2.839	−0.610	0.550
Adjusted model	0.581					0.001**
Groups		−0.008	−2.734	2.601	−0.053	0.958
Sex		0.097	−2.558	4.646	0.618	0.546
Pre‐intervention ADS score		0.789	0.423	1.047	5.020	<0.001***

*Note*: Hierarchical multiple regression analysis. Model with post‐intervention ADS score as dependent variable. The first step crude model included group as an independent variable, while the second step adjusted model included sex and pre‐intervention ADS score.

Abbreviation: ADS, Attitudes Toward Dementia Scale.

** *p* < 0.01

*** *p* < 0.001.

### Dementia Knowledge Scale

The DKS scores of the intervention group changed from 13.1 ± 1.1 to 12.3 ± 1.7, while those of the control group changed from 12.9 ± 2.0 to 13.3 ± 1.2. There was no main effect (*F* value = 0.256, *p* = 0.62) and no interaction effect (*F* value = 1.561, *p* = 0.22) (Table 2). The results of the hierarchical multiple regression analysis are presented in Table 4. In the first step, groups were not associated with post‐intervention DKS score (*β* = ‐0.342, *p* = 0.550). In the second step, the pre‐intervention DKS score was significantly associated with the post‐intervention DKS score (*β* = 0.618, *p* = 0.016). The results of the Wilcoxon signed rank sum test showed a small effect size for participants overall (*r* = −0.11), a moderate effect size for the intervention group (*r* = −0.32), and a small effect size for the control group (*r* = −0.22).

**Table 4:** Hierarchical multiple regression analysis of online clinical practice using VR or 2D monitors for predicting DKS scores after intervention.

Predictor variables	Adjusted *R* ^2^	*β*	95% CI for B		*t*	*p*‐value
			Lower	Upper		
Crude model	0.065					0.151
Groups		−0.342	−2.485	0.418	−1.502	0.550
Adjusted model	0.291					0.049*
Groups		−0.335	−2.307	0.284	−1.665	0.117
Sex		−0.304	−3.281	0.767	−1.324	0.205
Pre‐intervention DKS score		0.618	0.136	1.117	2.720	0.016*

*Note*: Hierarchical multiple regression analysis; Model with post‐intervention DKS score as dependent variable. The first step crude model included group as an independent variable, while the second step adjusted model included sex and pre‐intervention DKS score.

Abbreviation: DKS, Dementia Knowledge Scale.

* *p*  < 0.05.

### Images of the Elderly with Dementia

The IED score of the intervention group changed from 40.7 ± 6.1 before the intervention to 48.5 ± 6.5 after the intervention, while the score of the control group changed from 36.9 ± 6.5 to 47.6 ± 8.0, indicating the main effect of the intervention (*F* value = 39.029, *p* < 0.001) and no interaction effect (*F* value = 0.422, *p* = 0.52) (Table 2). The results of the hierarchical multiple regression analysis are shown in Table 5. In the first step, groups were not associated with post‐intervention IED score (*β* = −0.069, *p* = 0.780). In the second step, the pre‐intervention IED score was significantly associated with the post‐intervention IED score (*β* = 0.691, *p* = 0.004). The results of the Wilcoxon signed rank sum test showed a large effect size for participants overall (*r* = −0.88), a large effect size for the intervention group (*r* = −0.64), and a moderate effect size for the control group (*r* = −0.61).

**Table 5:** Hierarchical multiple regression analysis of online clinical practice using VR or 2D monitors for predicting IED scores after intervention.

Predictor variables	Adjusted *R* ^2^	*β*	95% CI for B		*t*	*p*‐value
			Lower	Upper		
Crude model	−0.054					0.780
Groups		−0.069	−7.977	6.088	−0.283	0.780
Adjusted model	0.352					0.001**
Groups		0.209	−3.149	8.910	1.018	0.325
Sex		−0.402	−15.593	0.433	−2.016	0.062
Pre‐intervention IED score		0.691	0.276	1.245	3.349	0.004**

*Note*: Hierarchical multiple regression analysis; Model with post‐intervention IED score as dependent variable. The first step crude model included group as an independent variable, while the second step adjusted model included sex and pre‐intervention IED score.

Abbreviation: IED, Images of the Elderly with Dementia.

** *p* < 0.01.

### System Usability Scale

The SUS was assessed for both groups after the online clinical practice. There were no significant differences between the intervention group (median: 70.0 [61.875−77.5]) and the control group (median: 55.0 [42.5−65.0]) (Mann–Whitney *U*‐test; *p* = 0.065). However, the intervention group was rated “good” and the control group “fair” according to the adjective rating criteria.^10^ The intervention group was rated one level higher than the control group.

### Class satisfaction (Q1–Q12) (Figure 3)

**Figure 3:**
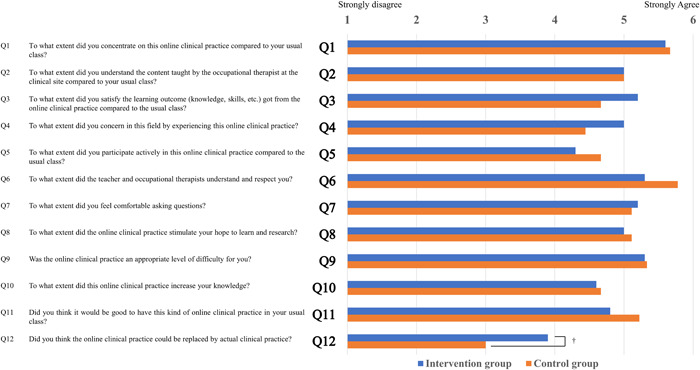
Results of practicum satisfaction assessment.

There were no significant differences between the two groups for Q1 to Q11 on the class satisfaction questionnaire. Q12 “Do you think the online clinical practice could be replaced by actual clinical practice?” tended to be higher in the intervention group (median: 4.0 [4.0–4.0]) than in the control group (median: 3.0 [3.0–4.0]) (Mann–Whitney *U*‐test; *p* = 0.095) (Figure 3).

### The results of the free description (Figure 4)

**Figure 4:**
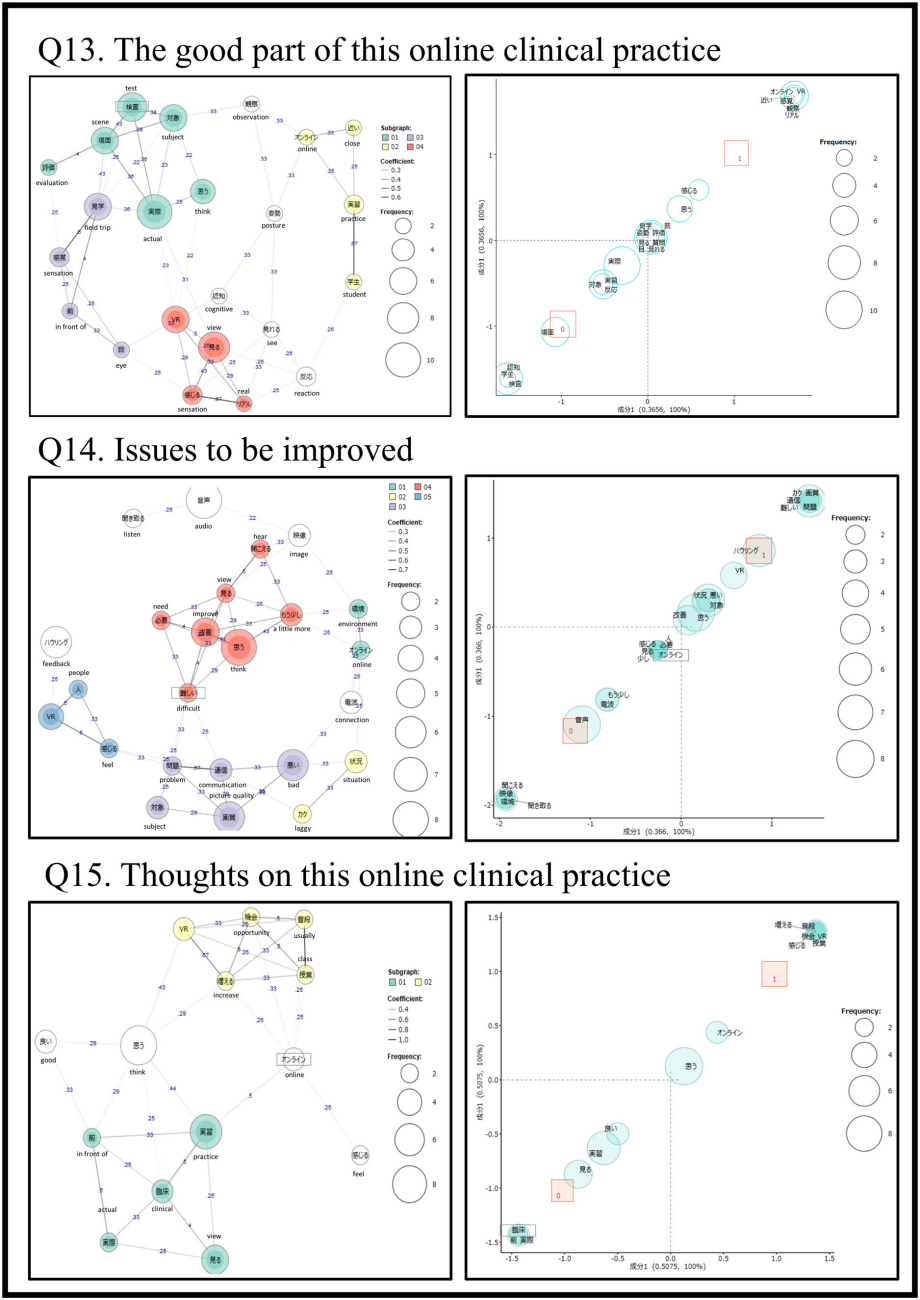
The results of free descriptions in three items of the class satisfaction questionnaire, quantitative text analysis using the KH coder (the left side is co‐occurrence network, the right side is correspondence analysis).

In Q13 “Which were good points in this online practice?”, a co‐occurrence network analysis was performed on the free descriptions of all participants, and four clusters were extracted. Each cluster included “test, evaluation, scene, subject, actual, think,” “online, close, practice, student,” “observation, sensation, in front of, eye,” and “VR, view, sensation, real.” These four clusters could be interpreted as (1) being able to observe an actual therapy (test) scene; (2) even though it was online, it was close to actual clinical practice; (3) the feeling of being able to observe in front of my eyes; and (4) being able to see and feel more realistically with VR. Correspondence analysis revealed that “online, real, sensation, close” were extracted as feature words in the intervention group. In the control group, “test, student, cognition, scene” were extracted. Common words in both groups were “observation, attitude, evaluation, question, and so on” were extracted. In other words, from these results, the characteristic words of the intervention group were extracted as “realistic” compared to the control group.

In Q14 “What could be improved about the online clinical practice?”, the co‐occurrence network analysis was performed, and five clusters were extracted. Each cluster included “online, environment,” “laggy, situation,” “image quality, bad, subject, communication, problem,” “think, improve, a little more, hear, view, need, difficult,” and “VR, people, feel.” That is, from these five clusters, (1) would like to see a little more improvement in the online environment; (2) the VR images were laggy; (3) specific problems with image quality, and poor communication conditions; (4) overall improvement; and (5) problems with feedback when conversing in VR (noise), could be interpreted. Correspondence analysis revealed that “image quality, laggy, communication, problem, feedback” were extracted as feature words in the intervention group. In the control group, “hearing, image, environment, listening” were extracted. Common words in both groups included “improvement, connection, bad, need, and so on” were extracted. The intervention group has a unique problem in that it highlights the problems of the device, such as laggy images. This was true for both groups and could be interpreted as indication that the online communication environment had a significant impact.

In Q15 “Overall impression of this online clinical practice?”, co‐occurrence network analysis was performed, and two clusters were extracted. Each cluster included “practice, view, clinical, actual, in front of” and “VR, opportunity, increase, usually, class.” That is, from these two clusters, (1) would be good to increase both online and VR in regular classes, and (2) would be good to be able to see online situations before clinical practice, could be interpreted. Correspondence analysis revealed that “VR, opportunity, increase, class” were extracted as feature words in the intervention group. In the control group, “clinical, pre, actual” were extracted.

## DISCUSSION

In this study, we investigated the effects of real‐time online clinical practice using real‐time VR compared with 2D PC screening on reducing stigma toward dementia, as measured by ADS and IED, in occupational therapy students. The main finding of this study is that the stigma toward dementia was immediately reduced in both the intervention and control groups. However, there was no difference between real‐time VR and 2D PC screening. This differed from our hypothesis that real‐time online clinical practice using real‐time VR would reduce dementia stigma more than 2D online practice. The secondary finding is that the knowledge assessed by DKS was not improved by this intervention.

### Attitudes Toward Dementia Scale, Dementia Knowledge Scale, and Images of the Elderly with Dementia

Although we hypothesized that real‐time VR would be more effective than 2D, as a result, the scores for the ADS and IED were improved in both groups and there was no interaction effect between the two groups. In other words, both types of real‐time VR and 2D online clinical practice could reduce the stigma toward dementia. Bacsu et al.^17^ found that education, contact, and mixed interventions were important to reduce dementia‐related stigma, with particular emphasis on introductions to the achievements of people with dementia, building relationships and engaging in purposeful learning. This suggests that knowledge about dementia and opportunities for mutual communication with a person with dementia can be effective in reducing stigma toward dementia. Yamaguchi et al.^18^ reported that direct contact is effective in improving attitudes compared with indirect contact with the observed person, such as recorded video‐based contact. In this study, although the online clinical practice was short, real‐time observation and communication with the observed subjects and occupational therapists may have provided sufficient opportunities for the target students to experience contact, to be educated, and to understand the personality of the person with dementia. This would lead to a better understanding of dementia and a change in attitude in both groups regardless of the type of intervention. The control group also had the same real‐time observation and communication as the intervention group. The ADS and IED used in this study changed before and after the intervention, suggesting that stigma reduction was accurately captured. Both assessments were easy to carry out, indicating that they could be useful in future large‐scale studies.

The secondary finding is that the participants' knowledge, as assessed by the DKS, was not improved by this intervention. Although it has been suggested that knowledge acquisition improves attitudes toward dementia, the DKS scores of the students as subjects in this study were very high at baseline and show almost a ceiling effect. In other words, it is possible that the students had already acquired sufficient knowledge, even though they had no prior exposure. Therefore, there would be no change in knowledge, but only in attitude.

### System Usability Scale

The SUS results also showed that the real‐time VR headset tended to be more usable than the 2D PC monitor in terms of the usability of the device. The high usability indicates the possibility that students can operate and use VR devices themselves, suggesting that there is little risk that VR education may increase the burden on educators and instructors. In addition, no students complained of physical discomfort, such as VR sickness, during the implementation of this study. These results suggest that online clinical practice using VR is safe and feasible without increasing the burden on educators. The real‐time VR is characterized by a 360‐degree image synchronized with the wearer's movements and the blocking of the landscape outside the head‐mounted display. This provides the subjective experience of being in one place or environment, even when one is physically situated in another.^19^ The immersive experience brought the students closer to the experience of undergoing clinical practice in a clinical setting, which may have resulted in pseudo‐direct contact with a person with dementia. Based on the above, it is suggested that real‐time VR has high usability, and the experience is similar to actual clinical practice due to the amount of information provided by the 360‐degree images and the immersive realistically feeling of being in the clinical site.

### The results of the questionnaire and text mining

The questionnaire and text‐mining results showed that real‐time VR brings a realistic feeling of clinical experience and that the participants hoped to incorporate it into their usual classes. However, some students were not comfortable with real‐time VR for reasons of poor video quality and audio. The discomfort interferes with concentration on the class, and students may miss the video and audio of important clinical situations. The immersive experience demonstrated the potential to bring students closer to the experience of clinical practice in the clinical setting, but future developments in technology are important. Therefore, it would be recommended to choose whether to use a 2D monitor or a real‐time VR headset depending on the Internet connection speed and teaching style.

Real‐time online clinical practice has the following strengths: (1) students can participate from anywhere, and (2) many students can participate immediately. Online clinical practice reduces the time and cost of traveling to the target facility. Observing rehabilitation scenes by many people is difficult due to space problems. Online clinical practice solves these problems. Therefore, online clinical practice, whose main purpose is to provide students with an opportunity to observe, can reduce the physical burden on students and cooperating facilities.

### Limitations

We should acknowledge several limitations of our study. First, the intervention and control groups received online clinical practice in close proximity due to the university's Internet communication environment. The setting prevented any blinding of participants, intervention implementers, and outcome assessors, and knowledge of group allocation and witnessed intervention risk bias of study results. Because the SUS results showed the feasibility of having students operate the VR equipment themselves and participate in the online clinical practice, it would be preferable to have each student participate from his or her own home when conducting a large‐scale study. This would allow the blinding of participants, intervention providers, and outcome assessors and avoid bias. Second, the sample size in this pilot study was small, as the target students were only third‐year undergraduates (10 in the intervention group, nine in the control group), and the statistical power of the two‐way analysis of variance may not have been sufficient. Therefore, the results of examining differences between the intervention and control groups should be considered with caution. Third, only one elderly patient with dementia (observed subject) participated in the online clinical practice. As the observed subject has mild dementia, the effect of observing people with advanced dementia is unclear from the results of this study. The effect of observing people with different types of dementia online should be investigated as a future study.

## CONCLUSION

This study demonstrated the feasibility of real‐time online clinical practice including real‐time VR and the appropriateness of the assessment choices, but also identified difficulties related to conducting a large‐scale study, such as issues related to video and audio quality, transmission speed, and difficulties in blinding of participants and intervention implementers. Also, this study shows that real‐time, online clinical practice experiences, including real‐time VR, can positively change attitudes toward dementia. Online clinical practice can be experienced at a distance, and a large group of people can experience the same scene. Contrary to our hypothesis, the VR online clinical practice in this study did not differ from the online practice using a 2D monitor in terms of improved attitudes and images. However, real‐time VR would have higher usability, and the experience was more similar to actual clinical practice than a 2D PC screen due to the amount of information provided by the 360‐degree images and the sense of immersion. In future large‐scale clinical trials, the environment in which students participate in online clinical practice should be devised to ensure an appropriate communication environment and complete blinding.

## AUTHOR CONTRIBUTIONS

K.U., H.T., M.U., R.I., K.Y., and Y.N. designed the experiment. K.U., H.T., K.N., M.U., A.T., and K.Y. provided environmental design and equipment support during the intervention. A.T. provided occupational therapy for the observed subject. Data analysis was performed by K.U., H.T., R.I., K.Y., and Y.N. The main manuscripts were written by K.U., H.T., M.U., R.I., and Y.N. All authors approved the final version of the manuscript.

## CONFLICT OF INTEREST STATEMENT

This study was supported by a grant from Daikin Industries, Ltd. Co‐researcher K.N. received this collaborative research grant from Daikin Industries, Ltd. Ryouhei Ishii is an Editorial Board member of *Psychiatry and Clinical Neurosciences Reports* and a co‐author of this article. To minimize bias, they were excluded from all editorial decision‐making related to the acceptance of this article for publication.

## ETHICS APPROVAL STATEMENT

This study was conducted with the approval of the Osaka Metropolitan University Graduate School General Rehabilitation Studies Ethics Committee (2022‐207).

## PATIENT CONSENT STATEMENT

Before participating in this study, all participants were informed orally and in writing about the purpose, content, and handling of personal information of the study, and gave their signed consent.

## CLINICAL TRIAL REGISTRATION

Trial registration information for this study is available at the following URL: https://center6.umin.ac.jp/cgi-open-bin/ctr/ctr_view.cgi?recptno=R000055774.

## Data Availability

The participants of this study did not give written consent for their data to be shared publicly, so due to the sensitive nature of the research, supporting data are not available.

## References

[1] Thornicroft G , Sunkel C , Alikhon Aliev A , Baker S , Brohan E , el Chammay R , et al. The Lancet Commission on ending stigma and discrimination in mental health. Lancet. 2022;400(10361):1438–1480. 10.1016/S0140-6736(22)01470-2 36223799

[2] Batsch N , Mittelman M . World Alzheimer Report 2012: overcoming the stigma of dementia. Available from: http://www.alz.co.uk/research/WorldAlzheimerReport2012.pdf

[3] Corrigan PW , Penn DL . Lessons from social psychology on discrediting psychiatric stigma. Am Psychol. 1999;54(9):765–776. 10.1037//0003-066x.54.9.765 10510666

[4] McCutcheon K , Lohan M , Traynor M , Martin D . A systematic review evaluating the impact of online or blended learning vs. face‐to‐face learning of clinical skills in undergraduate nurse education. J Adv Nurs. 2015;71(2):255–270. 10.1111/jan.12509 25134985

[5] Pei L , Wu H . Does online learning work better than offline learning in undergraduate medical education? A systematic review and meta‐analysis. Med Educ Online. 2019;24(1):1666538. 31526248 10.1080/10872981.2019.1666538 PMC6758693

[6] Tanaka H , Ueno K , Urakawa M , Naito Y , Ishii R . A preliminary approach to online clinical practicum in psychiatric day care. Rehabil Kyoiku Kenkyu. 2022;28:227–228.

[7] Li L , Yu F , Shi D , Shi J , Tian Z , Yang J , et al. Application of virtual reality technology in clinical medicine. Am J Transl Res. 2017;9(9):3867–3880. 28979666 PMC5622235

[8] Tang YM , Chau KY , Kwok APK , Zhu T , Ma X . A systematic review of immersive technology applications for medical practice and education—trends, application areas, recipients, teaching contents, evaluation methods, and performance. Edu Res Rev. 2022;35:100429. 10.1016/j.edurev.2021.100429

[9] Omori K , Shigemoto N , Kitagawa H , Nomura T , Kaiki Y , Miyaji K , et al. Virtual reality as a learning tool for improving infection control procedures. Am J Infect Control. 2023;51(2):129–134. 10.1016/j.ajic.2022.05.023 35659561

[10] Wu B , Yu X , Gu X . Effectiveness of immersive virtual reality using head‐mounted displays on learning performance: a meta‐analysis. Br J Educ Technol. 2020;51(6):1991–2005. 10.1111/bjet.13023

[11] NTT Communications . AVA.T.OUR. Available from: https://www.ntt.com/business/services/avatour.html

[12] Kim K , Kuroda K . Factors related to attitudes toward people with dementia: development attitude toward Dementia Scale and Dementia Knowledge Scale. Bull Soc Med. 2011;28(1):43–55.

[13] Bangor A , Kortum P , Miller J . Determining what individual SUS scores mean: adding an adjective rating scale. J Usability Stud. 2009;4(3):144.

[14] Higuchi K . New quantitative text analytical method and KH coder software. Jpn Sociolog Rev. 2017;68:334–350.

[15] Takeuchi Y , Kato M , Kitamura T , Toda D , Taniguchi Y , Shogenji M , et al. Development of professional care program for nurses in dementia wards and its educational effects. Am J Alzheimer's Dis Other Dementiasr. 2020;35:153331752095092. 10.1177/1533317520950925 PMC11005323 32865422

[16] Sasaki D . Analysis of the attitude within Asia‐Pacific countries towards disaster risk reduction: text mining of the official statements of 2018 Asian Ministerial Conference on disaster risk reduction. J Disaster Res. 2019;14(8):1024–1029. 10.20965/jdr.2019.p1024

[17] Bacsu JR , Viger M , Johnson S , McIntosh T , Jeffery B , Novik N , et al. Interventions to reduce stigma of dementia: findings from a scoping review. Innov Aging. 2019;3(1):S463. 10.1093/geroni/igz038.1729

[18] Yamaguchi S , Mino Y , Uddin S . Strategies and future attempts to reduce stigmatization and increase awareness of mental health problems among young people: a narrative review of educational interventions. Psychiatry Clin Neurosci. 2011;65:405–415. 10.1111/j.1440-1819.2011.02239.x 21851449

[19] Witmer BG , Singer MJ . Measuring presence in virtual environments: a presence questionnaire. Presen Teleoper Virt Environ. 1998;7(3):225–240.

